# The Micronaut–AM antifungal susceptibility testing method does not overestimate amphotericin B resistance in *Candida auris*

**DOI:** 10.1128/spectrum.00490-24

**Published:** 2024-04-05

**Authors:** Paraskevi Mantzana, Efthymia Protonotariou, Georgios Meletis, Areti Tychala, Lemonia Skoura

**Affiliations:** 1 Department of Microbiology, AHEPA University Hospital, Thessaloniki, Greece; Institut Pasteur, Paris, France

**Keywords:** *Candida auris*, amphotericin B, susceptibility testing

## LETTER

We read with interest the article by Siopi et al. (1) in which the authors pointed out the problem regarding the overestimation of amphotericin B (AMB) resistance in *Candida auris* by the Sensititre YeastOne (SYO) (Thermo Fisher Scientific, USA) antifungal susceptibility testing (AFST) method. Indeed, since June 2022, the Clinical and Laboratory Standards Institute (CLSI) updated that AMB susceptibility results should be interpreted with caution since the minimum inhibitory concentration (MIC) values for *C. auris* vary significantly among various testing methods used (2). European Committee on Antimicrobial Susceptibility Testing (EUCAST) and CLSI broth microdilution methods are the gold standards for susceptibility testing of *Candida* species; nevertheless, they are time consuming and labor intensive for the routine laboratory practice. So far, the only two available commercial colorimetric broth microdilution systems are SYO and Micronaut–AM (M-AM) (Bruker Daltonics GmbH & Co. KG). Up to now, very few data on MICs for *C. auris* isolates to AMB with the M-AM have been reported (3).

We studied the susceptibility profile of 89 *C*. *auris* isolates to AMB using M-AM collected from individual patients hospitalized in AHEPA University Hospital during December 2022 and October 2023. Most isolates were detected through active surveillance, including combined axillary/groin swabs, since the first *C. auris* case emerged in December 2022 in our hospital. A total of 57 surveillance for skin swab, 18 urine, 8 blood, 3 wound, 2 catheter, and 1 respiratory cultures were included. Identification to the species level was performed by matrix-assisted laser desorption/ionization time-of-flight mass spectrometry (MALDI Biotyper Sirius IVD System, version 12.0; Bruker Daltonics GmbH & Co. KG). In the context of a regional surveillance program, selected strains were sequenced for both D1–D2 and ITS1 ribosomal regions for molecular confirmation and multilocus phylogenetic analysis, which revealed that the isolates of our hospital cluster with the South Asian (clade I) strains according to the local and national epidemiology (unpublished data). AFST for M-AM was performed according to the manufacturer’s instructions. In parallel, AFST was tested with the automated system Vitek2 (bioMérieux, France) for 66 of 89 isolates. Both MIC_50_ and MIC_90_ for AMB were 1 (MIC range from 0.125 to 1.0) using M-AM, whereas those using Vitek2 were 8 and 16, respectively (MIC range from 2 to >16). As there are currently no established *C. auris*-specific susceptibility breakpoints, the interpretation was done according to the epidemiological cutoff value of 2 mg/L, an MIC value proposed by both EUCAST and CLSI which discriminates the wild-type isolates (4) and the tentative MIC breakpoint of ≥2 mg/L according to Centers for Disease Control and Prevention guidelines (5). All studied strains were tested susceptible to AMB using M-AM, while all those tested with Vitek2 were found resistant. Our results are concordant with the falsely high values for AMB in *C. auris* isolates with Vitek2 that have already being pointed out (6). The susceptibility to AMB using M-AM coincides with the low levels for AMB resistance among *C. auris* isolates (4). The MIC range of M-AM (0.125–1.0) is consistent with the MICs observed using broth microdilution (7) and especially with the EUCAST broth microdilution method (0.25–1.0) (4). High rates of AMB resistance have been reported across different testing methodologies. It seems that the M-AM commercial broth microdilution testing method does not overestimate AMB resistance in *C. auris* as observed by Siopi et al. with SYO (1). This refers to clade I *C. auris* isolates and must be confirmed with the EUCAST broth microdilution reference method and the detection of resistance mechanisms.
